# Analyzing tourism reviews using an LDA topic-based sentiment analysis approach

**DOI:** 10.1016/j.mex.2022.101894

**Published:** 2022-11-05

**Authors:** Twil Ali, Bencharef Omar, Kaloun Soulaimane

**Affiliations:** Laboratory of Computer and Systems Engineering, Faculty of Sciences and Techniques, Cadi Ayyad University, Marrakesh 40000, Morocco

**Keywords:** LDA, Sentiment analysis, Online reviews, Topic modeling, Natural language processing, User generated content

## Abstract

It has become increasingly necessary to automate systems for organizing and classifying user reviews according to their domain-specific aspects and sentiment polarities, as online customer opinions have increased on specialized platforms and social networks. This study's methodology employs a combination of topic modeling and sentiment analysis, as well as human validation techniques of topic labels, to extract valuable insights about Marrakech city from TripAdvisor reviews. Through this technique, tourism practitioners and field specialists may dive deeper into online users generated content, leveraging aspect-based sentiment analysis to explore each destination's weaknesses and strengths.

• Data collection and pre-processing.
• Extracting latent topics using LDA algorithm (Latent Dirichlet Allocation) on collected reviews.
• Applying sentiment analysis to each topic.

## Graphical abstract

**Figure:**
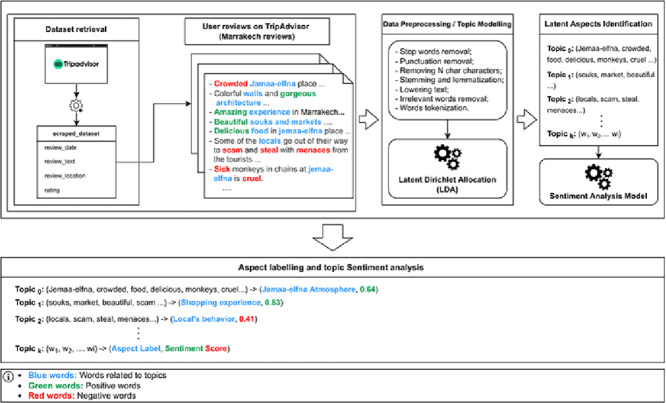

**Specifications Table**

**Table:** 

Subject Area	Computer Science
More specific subject area	*Natural Language Processing and Topic Modelling*
Method name	*LDA Topic-Based Sentiment Analysis approach.*
Name and reference of the original method.	*N/A*
Resource availability	• https://www.anaconda.com/products/individual • https://www.nltk.org/ • https://research.google.com/colaboratory/ • https://github.com/cjhutto/vaderSentiment • https://github.com/victor7246/jointtsmodel

## Method details

**Fig. 1:**
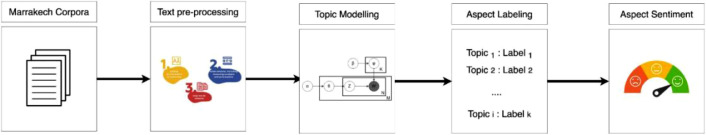
Methodological process for Topic-Based Sentiment analysis approach.

Based on earlier research, the nature of the study set forth was relatively new for the tourist sector in Morocco; hence, special considerations were taken into account to construct the approach. Collaborating with tourism experts was necessary to assist in analyzing TripAdvisor data. The primary goal of this technique is to combine a machine learning algorithm with tourism domain knowledge to extract meaningful insights from reviews and evaluate tourists' lived experiences in Marrakech.

(Fig. 1) Illustrates the methodological process for the proposed approach of Topic-Based Sentiment analysis. We presented our initial findings with a case study on more than 39,200 English reviews of Marrakech attractions on TripAdvisor. The data was scraped and preprocessed using Python language. We discuss the process used to create the results in the following sections.

## Related work

Topic-Based Sentiment Analysis involves extracting opinion targets (topic terms) and the sentiment expressed towards them. User feedback in product evaluations and other textual data is critical for manufacturers, merchants, and service providers [1]. Topic-Based sentiment analysis provides a basis for managing public online opinions and plays a critical role in identifying the popular subjects and sentiment shifts on the Internet. Aspect-based sentiment analysis of reviews is quite helpful for understanding user opinion at a fine-grained level; It is divided into two sub-tasks, First extracting aspects from each review and then categorizing aspect-based evaluations based on sentiment polarity [2]. Over the last decade, the research community has concentrated on the challenge of analyzing user opinions, with a particular emphasis on online consumer evaluations. *Customer opinion analysis* is a problem that may be broken into numerous subtasks, such as identifying the aspect (aspect classification) and detecting the opinion about the aspect of the product being assessed. Topic models have been used with Sentiment Analysis topics and sentiment of words [3,4].

It is common to use certain apriori information to bias the LDA hyperparameters during a topic modelling process. Usually, to model the polarity of the documents, seed words are carefully selected [5]. Otherwise, [6] aimed to explore the limits of open innovation by extracting evidence from user-generated content (UGC) on Twitter, using Machine Learning Sentiment Analysis algorithm and a mathematical topic modelling using Latent Dirichlet allocation algorithm to analyze the tweet corpora to explore the characteristics and sentiments of open innovation according to UGC from Twitter. In this connection, [7] Consider part-of-speech (POS) tags that might be utilized to express consumers’ opinions in online reviews as visual information in topic modelling and sentiment analysis, unlike the other topic modelling approaches. However, [8] used JST model, which uses only specific keywords extracted from the LDA model, which uses only a few keywords describing each aspect/sentiment without any labelled examples.

Few other works connected to our study; For instance, [9] used LDA on 100 reviews from the TripAdvisor platform to evaluate sentiment analysis and reading tendency from tourist reviews. [10] proposes sentiment attribution analysis with hierarchical classification and automatic aspect detection to improve social listening for active marketing and urge potential business optimization to revive the business from surviving to succeeding after the COVID-19 pandemic. Therefore, in this study, an unsupervised learning model topic model-based approach will be elaborated. This system aims to pinpoint hidden tourists' concerned topics and extract corresponding sentiment polarity simultaneously using lexicon-based sentiment analysis algorithms. It is expected to facilitate designers to obtain feature-level visitors' concerns from a large volume of textual review corpora. Otherwise, [11] Studied the aim of opportunities and challenges for remote work using digital technologies. The authors used computer-aided text analysis, natural language processing, and topic modelling on Twitter's user-generated contents to retrieve sentiments of the main underlying topics and challenges discussed among users.

## Dataset retrieval

**Fig. 2:**
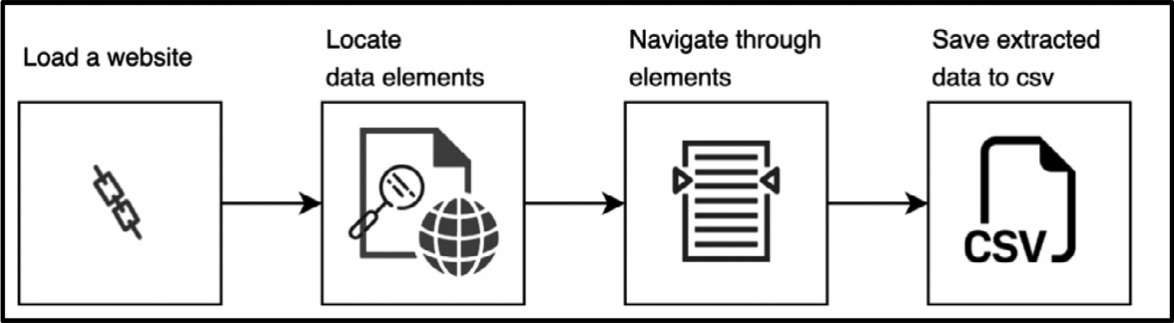
Data retrieval flowchart.

As a first step, we used a Python script based on the Selenium package to extract TripAdvisor reviews for various attractions in Marrakech over 12 years (2008 to 2020). More than 39,200 reviews were gathered, and the following fields are targeted: review date, user location, rating, and review text. Ratings 1 and 2 on TripAdvisor were assigned as Negative, rating 3 was assigned as Neutral, and ratings 4 and 5 were assigned as positive [12].

(Fig. 2) Illustrate the web scraping process. TripAdvisor's links crawled using the Selenium package and a web driver. The following section outlines the steps for scraping destination reviews. Although Python has many libraries that facilitate the scraping of websites, Selenium is the best tool for dealing with interactive travel websites. In order to extract review data from TripAdvisor, we begin by illustrating the scraping process.

- Step 1: Enter some TripAdvisor attraction's URL and use an HTTP request to access the URL using a web driver

The target URL for TripAdvisor contains three main components:

“https://www.tripadvisor.com/Hotel_Review-g293734-d23205490-Reviews-or5-Les_Jardins_de_Marrakech-Marrakech_Marrakech_Safi.html”

• *Attraction code: “g293734-d23205490”* Special code designs each attraction in the TripAdvisor URL.
• *Page number: “or5”* Each page on TripAdvisor contains five reviews, every time we change the page, the number associated with *“or”* increases accordingly (Page 1: "or5", Page 2: "or10"...), which allows scraping over all the pages.
• *Attraction name: “Les_Jardins_de_Marrakech”* TripAdvisor's attraction name separated by underscores.

**Fig. 3:**
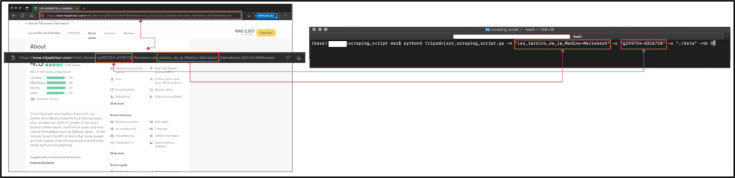
Define the target link for scraping.

In order to facilitate the scraping process, the script uses only these three parameters as arguments instead of the full link (Fig. 3).

**Fig. 4:**
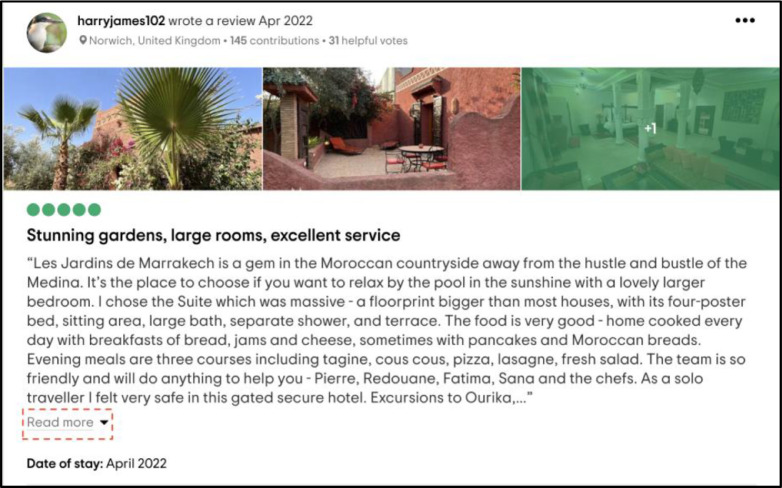
Example of an interactive web page.

Other Python libraries (BeautifulSoup, Request, Scrapy) allow scraping everything pictured on the web page. Despite this being good news, hidden information cannot be scraped until we click on it. Other Python libraries, such as BeautifulSoup, request, and Scrapy, cannot scrape the contents hidden behind the *"Read more"* button, as shown in (Fig. 4).

The majority of interactive travel websites use JavaScript rather than Hypertext Markup Language (HTML) or CSS, which enables interactive functionality. The core functionality of JavaScript differs from many other programming languages in that it enables dynamic and interactive web pages. In order to emulate human browsing behaviour on sites that heavily use JavaScript, we must write code in a manner that emulates human behaviour.

- Step 2: Locate review elements using the web driver and XPath syntax

*- WebDriver:* WebDrivers exist for most modern internet browsers. When executing the script, a browser window will open on and perform the actions specified in the scraping code [13]. In the following section, We assume that the driver used is chrome-driver for google chrome navigator. The simplest method to use the WebDriver is to put it in the same directory as the scraping Python scripts; Once the WebDriver opens the browser, we can interact with the browser until it is closed.

**Fig. 5:**
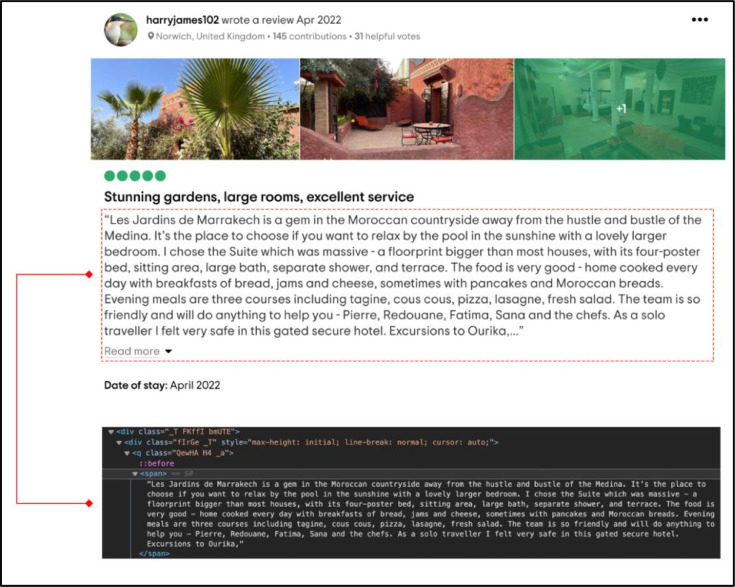
Example of review's HTML structure.

- *XPath:* [14] Once WebDriver opens a webpage, we aim to extract the content shown on the screen and save it . We must first examine the source code of the webpage components we wish to scrape in order to collect this data. The most straightforward approach to examining this information is to right-click on the section of the website we want to scrape and choose "Inspect”. As an example, suppose the component of the HTML code written to showcase the reviewer's comment is shown in (Fig. 5), in which the <span>../span> encloses a generic inline container for phrasing content chosen when displaying the reviewer's comment.

- Step 3: Crawl over the web page contents using the Selenium package functionality "find_elements_by_xpath”

**Fig. 6:**
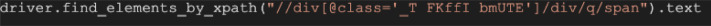
Example of XPath expression.

To extract the information from the HTML code (Fig. 5), the XPath has to be written as //div[@class='_T FKffI bmUTE']/div/q/span, which means getting the current node (//) with the tag <div> where the class attribute contains the value "_T FKffI bmUTE" (Fig. 6); However, often this value is very long and contains multiple special characters.

- Step 4: Loop each element into a DataFrame and save it to a CSV file.

Although most scraping operations can be performed using the abovementioned tools and XPath expression language, one concern requires special attention. As seen in (Fig. 4), many TripAdvisor comments are long, requiring users to click the *"Read more"* button to read the entire comment. Researchers must be able to click this button to scrape complete reviews automatically. Selenium provides a set of "actions" that the browsers may do for this purpose, such as clicking items. Using a "try-except" statement is advised to avoid stopping the script if one review is not expandable.

**Fig. 7:**
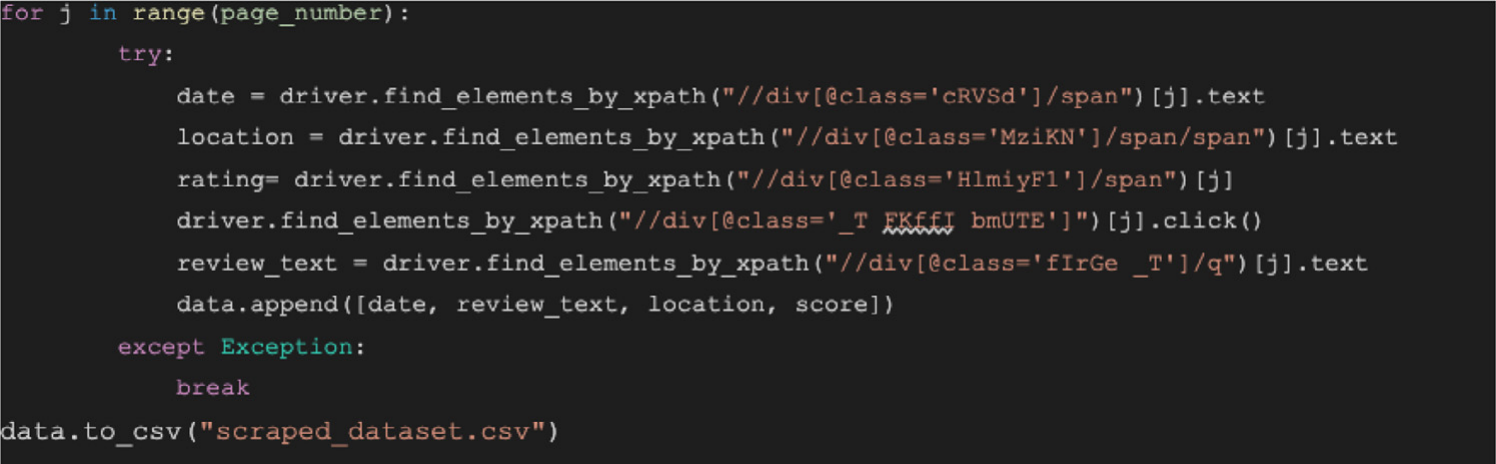
Python Script to Retrieve all the dataset elements.

Afterwards, after identifying all class names in the HTML source code, a for-loop over a specified number of pages (Fig. 3) can be used to store all the content and save it into a CSV file using the Python library Pandas (Fig. 7).

## Data pre-processing

Data preprocessing can refer to the manipulation or removal of data before it is used to ensure or improve performance. It is an important step in the data mining process [15]. This stage is, however, essential, and the quality of the analyzed data for conducting an aspect-based sentiment approach. Basic Natural Language Processing methods were used to pre-process the data, such as:

• Removing punctuation from reviews: This step involves removing all punctuation from the text. In Python «string» library, there is a predefined list of punctuation marks, such as '!"#$%&'()*+,-./:;?@[\]^_`{|}∼.'

For example, a raw review seemed as:

*"A market that lights up at night with local goodies. There are snake charmers (#NOT_a_fan !!!) and henna artists that force themselves on you !."*

After removing punctuations, the review became:

*" A market that lights up at night with local goodies There are snake charmers NOT a fan and henna artists that force themselves on you "*

• Stemming and lemmatization: Also known as the text normalization stage, in which words are reduced to their root or elemental form. A Porter stemmer package is used for stemming, which reduces inflectional and derived word forms to a common base form.

For example, the review:

*" A market that lights up at night with local goodies There are snake charmers NOT a fan and henna artists that force themselves on you "*

Will be stemmed to:

*" A market that light up at night with local goodi There are snake charmer NOT a fan and henna artist that forc themselv on you"*

• Lowering the reviews: A standard step in pre-processing is to convert the text to lowercase. For example:
  *" a market that lights up at night with local goodies there are snake charmers not a fan and henna artists that force themselves on you "*
• Removing N char characters: this task aims to pull a string of length 'n' from the review; in our methodology, we removed characters of 2 or less to keep only the words.
• Stop word removal: Stop words are generally removed from the text because they don't add any value to the analysis. These words have little or no meaning. The Python NLTK library contains a list of English stop words. There are several of them, such as: (i, me, my, myself, we, our, ours, ourselves, you, you're, you've, you'll, you'd, your). For example:
  *" a market that lights up at night with local goodies there are snake charmers not a fan and henna artists that force themselves on you "*

After removing stop words, the review became:

*" market lights night local goodies snake charmers fan henna artists force "*

• Irrelevant words removal: In addition to eliminating common stop words, eliminating irrelevant terms is a beneficial task in the pre-processing process, which involves eliminating noisy terms such as names of attractions, links, and proper nouns.

This step depends on the project and dataset's nature, and the list of these terms is usually built after being identified in the exploratory data analysis phase and the exploration of word frequencies.

For example:

*“ Our guide was Issmail and our driver was Mohamad - both were excellent all day long at Marrakech !*

*Highly recommend the day trip as Issmail knows the areas like the back of his hand and so his knowledge is excellent ”*

After applying text normalization steps, removing stopwords, and irrelevant words removal, the review became:

*“ guide driver excellent day long highly recommend day trip areas hand knowledge excellent ”*

Particularly during the data exploration phase [16]. Each review is tokenized into a collection of unigrams, and this lexical analysis is frequently utilized to discover those words and preserve the corpus free of noise and meaningless keywords [15].

Natural language processing (NLP) involves the transformation of User Generated Content (UGC) into a vector representation to function [17]. Text vectorization approaches, such as TF-IDF, Bag-of-Words, and vectorization, are widely used in classification algorithms and can enable converting textual input to numeric feature vectors. As a result, to quantify and transform the text into a numerical representation in documents, we compute a weight for each phrase representing the phrase's value in the document and corpus. The TF-IDF method is a popular approach in text mining and information retrieval.

(1) tfidf(t,D)=tf(t,D)·idf(t,D)

Where:

tf(t,d)=log(1+freq(t,d))

Inverse Document Frequency (IDF). IDF measures how important a term is. While computing TF, all words are considered to be extremely significant. It is acknowledged, however, that certain words, like "is," "of" and "that," can occur several times but have little relevance. Thus, we need to calculate the weight of rare words across all documents in the corpus. Terms that occasionally appear in the corpus have a high IDF score. It is given by equation (2).

(2) $$Score_{UMass\;}\left(w_{i\;},w_{j}\right)=log\left(\frac{1}{count\left(d\;\epsilon \;D:t\;\epsilon \;d\right)}\right)$$

## Topic modelling

Topic modelling refers to distinguishing topics that best describe a group of documents. These topics can solely emerge throughout the topic modelling process. A topic modelling analysis was carried out using the LDA algorithm [18] to discover aspects and specific challenges concerning tourist experience and sentiment in the research. The topic analysis allows tourism experts to gain more in-depth awareness of the latent themes that drive consumer sentiment and categorize these factors to make particular inferences from large sets of textual data [19]. This technique is used to find hidden dimensions in our corpus of reviews collected from TripAdvisor.

LDA topic modelling is a probabilistic model that needs a prior definition of the number of topics at the beginning, so in order to define the optimum number of topics, a crucial step is needed, which is the calculation of the coherence score [20]. Topic Coherence (3) measures the degree of semantic similarity between high-scoring terms within a single topic.

(3) $$CoherenceScore=\sum_{i<j}\cdot sc\left(w_{i},w_{j}\right)$$

$w_{i}$ = the top words of the topic.

There are two types of topic coherence scores:

• Extrinsic UCI measure:
  (4) $$Score_{UCI\;}\left(w_{i\;},w_{j}\right)=\frac{log_{p\;}\left(w_{i\;},w_{j}\right)\;}{\;P\left(w_{i\;}\right)P\;\left(w_{j}\right)}$$
• Intrinsic UMass measure:
  (5) $$Score_{UMass\;}\left(w_{i\;},w_{j}\right)=\frac{log_{D\;}\left(w_{i\;},w_{j}\right)\;+\;1}{\;D\left(w_{i\;}\right)}$$

## Latent Aspect Identification and Aspect labelling

We can distinguish between semantically interpretable topics and statistical artefacts using these metrics [21]. The model returned the correct number of dimensions; four topics were determined using this technique, followed by their terms and weights. The topics were then labelled based on experts’ interpretations; Overall, Topics were labelled as follows:

• Topic 1: Jamaâ-el-Fna atmosphere;
• Topic 2: Shopping experience;
• Topic 3: Citizen's behaviour;
• Topic 4: Overall touristic experience.

**Fig. 8:**
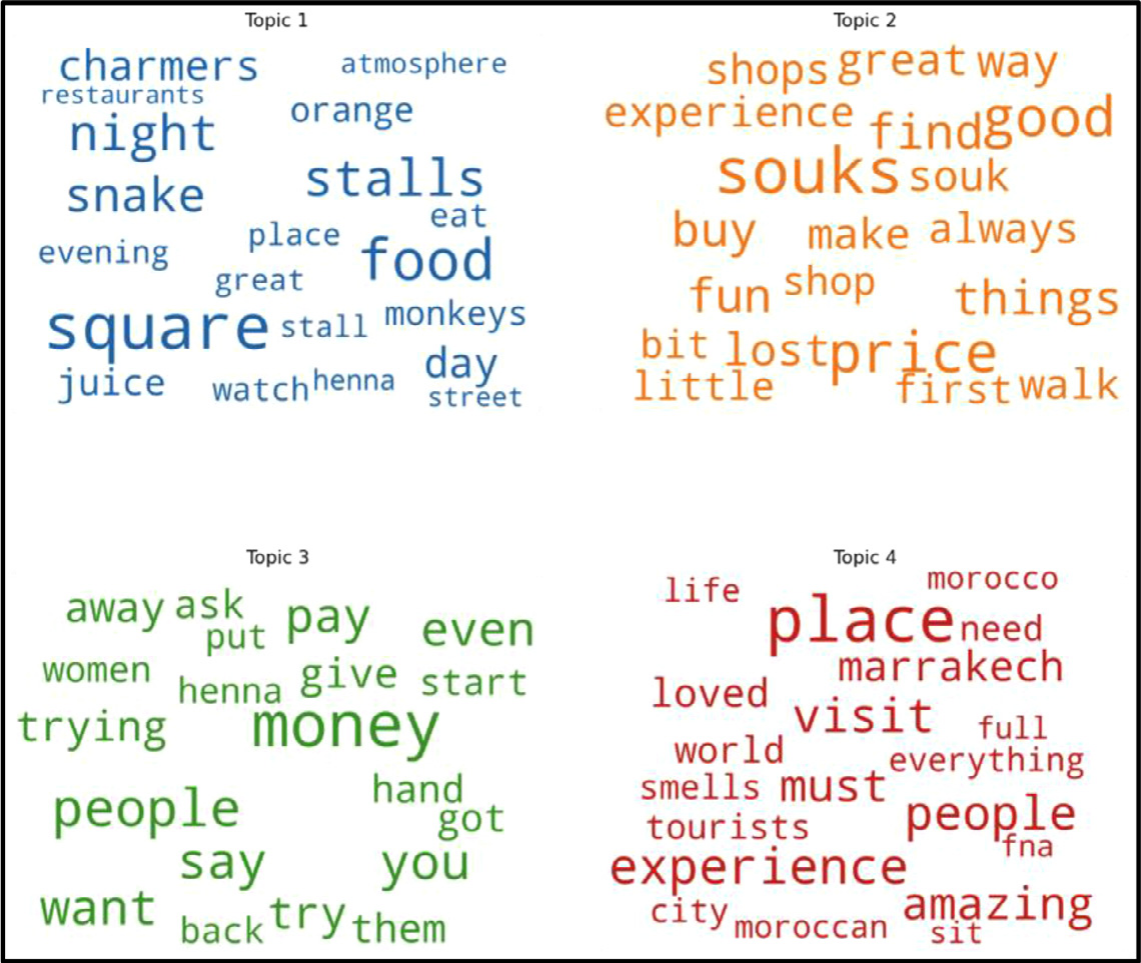
Representation of the underlying terms of each topic.

In this methodology, The Jamaâ-el-Fna label is distinguished by terms such as square, monkeys, charmers, food, and stall, accurately indicating the atmosphere of this place. The second dimension is the shopping experience, characterised by words such as souks, buy, pleasure, prices, and stores, which provides insight into the subject's reactions. The third dimension is citizen's behaviour, represented by terms such as people, money, try, ask, and henna, describing commercialism in the behaviour of the citizens. Finally, the last dimension is the description of the overall experience with terms that denote the pleasure and the overall feedback on their tourist experience, such as experience, must, visit, amazing, loved, and everything (Fig. 8).

## Topic sentiment analysis

Sentiment analysis aims to identify opinions, emotions, and evaluations expressed in natural language [22]. The main objective is to predict the sentiment orientation (i.e., positive, neutral, or negative) by analysing the sentiment or opinion words and expressions in sentences and documents; There have been many studies on this topic recently.

This approach involves analysing extracted topic words through a lexicon-based sentiment analysis approach to identify the emotional state associated with each dimension.

As a result, the authors analysed the corpora of each dimension to determine what tourists like, what they appreciate, and what their primary concerns are; VADER [23] and TextBlob^1^ lexicon rule-based algorithms were examined. VADER algorithm scored highest in comparison to TripAdvisor's bubble rating system. After distinguishing the fundamental dimensions and calculating the dataset's sentiment score, a topic-based opinion examination was conducted to see each topic's estimation extremity. Even though the negative reviews are seen as more critical to affecting tourists [24].

(6) $$Topic\;valence=\;\frac{Actual\;Number\;of\;Positive\;Reviews\;-\;Expected\;Number\;of\;Positive\;ReviewsReviews}{Total\;reviews\;of\;the\;topic}$$

Calculating rational dimension valence helps evaluate whether a topic is positive or negative. Each category of attraction has its own set of topics [25]. The cross-tabulation technique determines each topic's valence (6); the comparison indicates the positive reviews and the observed positive ones. The predicted positive review is calculated by adding the total number of positive reviews in the dataset to the total number of reviews in each dimension and dividing by the total number of reviews (Table 1).

**Table 1:** Summary of results of Joint Topic Model.

Jamaâ-el-Fna atmosphere		Shopping experience		Citizen's behaviours		Overall experience	
Good	Bad	Good	Bad	Good	Bad	Good	Bad
Swallowers	Evolves	Discount	Overprice	Freebies	Demanding	Powerful	Populated
Teller	Malders	Compromise	Antique	Offering	Troubles	Wonderful	Overrated
Acrobats	Smoke	Reduce	Bargaining	Ample	Grasp	Equipped	Loathe
Charmers	Strings	Price	Overpaid	Compassion	Protests	Cultured	Disrespect
BBQs	Haze	Purchase	Wasting	Befriend	Bothering	Legendary	Facade

Using the studied dataset, the authors performed quantitative and qualitative comparisons of the Joint Topic Model approach for sentiment analysis [3] versus the proposed model. To maintain consistency between the two approaches, the authors used the exact keywords from the output of the LDA model.

Using the TripAdvisor bubble scoring system and lexicon-based algorithm scores, the overall sentiment of the site was analyzed. According to TripAdvisor's classification system, reviews with scores 1 and 2 are negative, reviews with scores 3 and 4 are neutral, and reviews with scores 4 and 5 are positive. As shown in Table 2, TripAdvisor reviews indicated 74.4% positive, and TextBlob scores indicated 84.5% positive. However, VADER Sentiment's sentiment analysis results were close to TextBlob's, with 83.3% observed positive reviews to 74.4% expected positive reviews.

**Table 2:** Summary of results of the topic sentiment using TextBlob and VADER sentiment.

Sentiment Model	Topic	Positive		Neutral		Negative	
		Observed	Expected	Observed	Expected	Observed	Expected
Textblob	Jamaâ-el-Fna atmosphere	11,344	10,698	496	1786	1296	650
	Shopping experience	13,968	12,312	426	2668	1760	1174
	Citizen's behaviors	3438	2204	136	1212	1432	1590
	Overall experience	4010	3968	264	512	646	442
Vader sentiment	Jamaâ-el-Fna atmosphere	11,670	10,698	336	1786	1128	650
	Shopping experience	13,828	12,312	312	2668	2014	1174
	Citizen's behaviors	3222	2204	76	1212	1708	1590
	Overall experience	4010	3968	264	512	646	442

The authors evaluated the accuracy of sentiment predictions at the subject level by comparing the sentiment predictions of our lexicon-based models with the sentiment predictions of the joint subject model (Table 3).

**Table 3:** Benchmark of Topic sentiment analysis results.

Sentiment analysis Model	Average Accuracy
TextBlob	77.3%
VADER Sentiment	72.6%
JST Sentiment	69.6%

(Table 3) shows the results obtained. On the same data set, the lexicon-based models outperform the JST sentiment model by 3% and 7.7%, respectively. These models excel at capturing general feelings about topics without the need for post-processing, which is what explains their good accuracy.

## Conclusion, limitations, and future research

As a soft aspect of service-dominated tourism planning, online feedback is a way to share information on online travel platforms [26]. As a result, text mining of user-generated content will help the tourism industry make smart and efficient decisions by leveraging the opinion of millions of tourists, comments, and conversations [27]. Thus, this study is designed to identify the underlying Topics and sentimental polarity reflected in online reviews. Four topics were identified using the topic modelling technique named Latent Dirichlet Allocation [18].

**Fig. 9:**
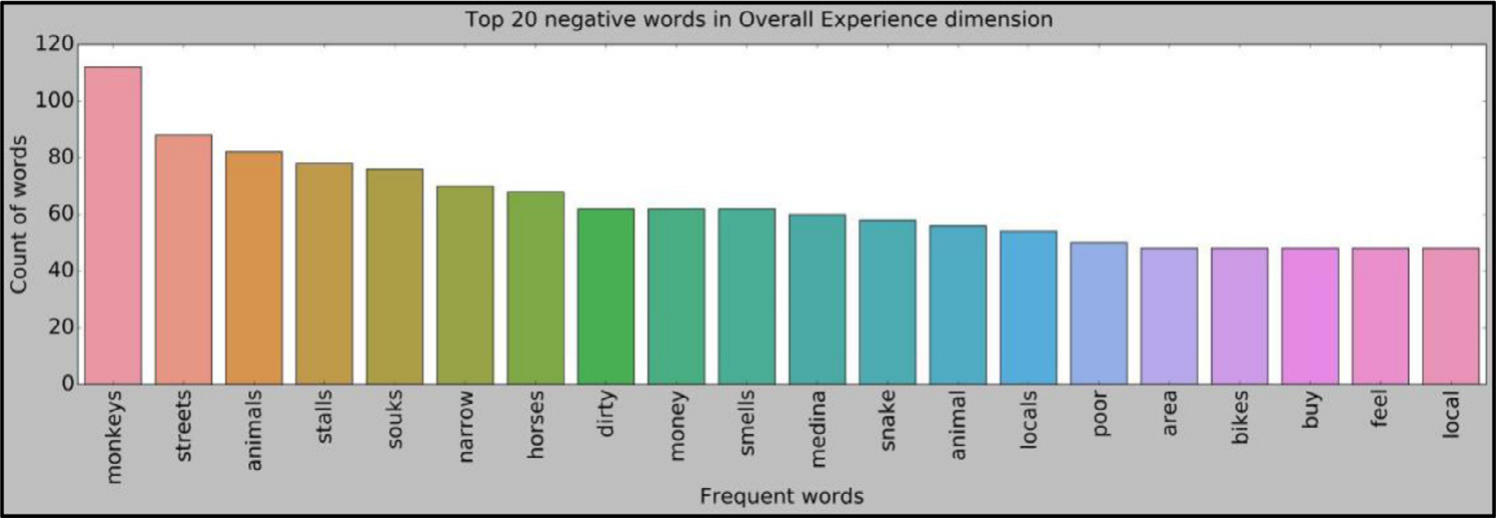
Representation of top 20 negative words.

Since the negative reviews will undoubtedly be of quality to taking suitable corrective measures, the reviews below each topic have been further examined to look for the fundamental reasons behind visitors’ dissatisfaction. However, the findings in this research provide a much better comprehension of tourist actions and managerial viewpoints that can boost the touristic experience in Marrakech (Fig. 9). Nevertheless, the present study has several limitations. First, the sentiment analysis algorithms used in this analytical framework are primarily rule-based, and these models do not recognize irony, sarcasm, and connotations [28]. Second, data may be destination-specific; therefore, results, such as sentiments, ratings, and aspects results in distribution, should be interpreted with caution for other in-domain applications. Also, the developed system focused only on English language reviews; future research can also explore other languages to gain more insights. Thus, the analytical framework developed in this research study might be extended to other services and industries. Future analysis could also consider using more machine learning or deep learning algorithms.

## Declaration of interests

The authors of this paper acknowledge that there is no conflict of interest in the publishing of this methodology and that permission was obtained to use the methodology and findings for research purposes.

## Data Availability

I have shared the code and data at the Attach File step.

## Appendix Supplementary materials

Image, application 1

## Footnotes

1 https://github.com/sloria/TextBlob.

## References

[1] Tun Thura Thet, Na J-C, Khoo CSG. (2010). Aspect-based sentiment analysis of movie reviews on discussion boards. J. Inf. Sci..

[2] Aspect-Based Sentiment Analysis for Polarity Estimation of Customer Reviews on Twitter n.d. https://techscience.com/cmc/v67n2/41318/html (accessed May 4, 2022).

[3] Joint sentiment/topic model for sentiment analysis | Proceedings of the 18th ACM conference on Information and knowledge management n.d. https://dl.acm.org/doi/abs/10.1145/1645953.1646003 (accessed May 4, 2022).

[4] García-Pablos A, Cuadros M, Rigau G. W2VLDA: Almost Unsupervised System for Aspect Based Sentiment Analysis. arXiv:1705.07687 Cs 2017.

[5] Paul MJ. (2015).

[6] Saura JR, Palacios-Marqués D, Ribeiro-Soriano D. (2022). Exploring the boundaries of open innovation: Evidence from social media mining. Technovation.

[7] Tang M, Jin J, Liu Y, Li C, Zhang W (2019). Integrating topic, sentiment, and syntax for modeling online reviews: a topic model approach. J. Comput. Inf. Sci. Eng..

[8] Huang J, Meng Y, Guo F, Ji H, Han J. Weakly-Supervised Aspect-Based Sentiment Analysis via Joint Aspect-Sentiment Topic Embedding n.d.:11.

[9] Putri I, Kusumaningrum R. (2017). Latent Dirichlet Allocation (LDA) for sentiment analysis toward tourism review in Indonesia. J. Phys..

[10] Win MN, Ravana SDR, Shuib L. (2022). Sentiment attribution analysis with hierarchical classification and automatic aspect categorization on online user reviews. Malays. J. Comput. Sci..

[11] Saura JR, Ribeiro-Soriano D, Zegarra Saldaña P. (2022). Exploring the challenges of remote work on Twitter users’ sentiments: from digital technology development to a post-pandemic era. J. Bus. Res..

[12] Xiang Z, Du Q, Ma Y, Fan W. (2017). A comparative analysis of major online review platforms: Implications for social media analytics in hospitality and tourism. Tour. Manag..

[13] García B, Delgado Kloos C, Alario-Hoyos C, Munoz-Organero M. (2022). Selenium-Jupiter: a JUnit 5 extension for Selenium WebDriver. J. Syst. Softw..

[14] Holman GK. (2002).

[15] Ali T, Marc B, Omar B, Soulaimane K, Larbi S. (2021). Exploring destination's negative e-reputation using aspect based sentiment analysis approach: case of Marrakech destination on TripAdvisor. Tour Manag. Perspect..

[16] Abel J, Teahan W. (2005). Universal text preprocessing for data compression. IEEE Trans. Comput..

[17] Ruelens A. (2022). Analyzing user-generated content using natural language processing: a case study of public satisfaction with healthcare systems. J. Comput. Soc. Sci..

[18] Blei DM. Latent Dirichlet Allocation n.d.:30.

[19] Ren G, Hong T. (2017). Investigating online destination images using a topic-based sentiment analysis approach. Sustainability.

[20] Newman D, Lau JH, Grieser K, Baldwin T. Automatic evaluation of topic coherence n.d.:9.

[21] Aletras N, Stevenson M. (2013).

[22] Liu B. Sentiment Analysis and Opinion Mining n.d.:168.

[23] C.J. Hutto EG. (2014).

[24] Fernandes T, Fernandes F. (2018). Sharing dissatisfaction online: analyzing the nature and predictors of hotel guests negative reviews. J. Hosp. Mark. Manag..

[25] Taecharungroj V, Mathayomchan B. (2019). Analysing TripAdvisor reviews of tourist attractions in Phuket, Thailand. Tour Manag..

[26] Girija S, Sharma DR, Kaushal V. (2022). Exploring dimensions of the customer experience at budget hotels during the COVID-19 pandemic: a netnography approach. Qual. Mark. Res. Int. J..

[27] Guan C, Hung Y-C, Liu W. (2022). Cultural differences in hospitality service evaluations: mining insights of user generated content. Electron. Mark..

[28] Meriem AB, Hlaoua L, Romdhane LB. (2021). A fuzzy approach for sarcasm detection in social networks. Procedia Comput. Sci..

